# Exploring Action Dynamics as an Index of Paired-Associate Learning

**DOI:** 10.1371/journal.pone.0001728

**Published:** 2008-03-05

**Authors:** Rick Dale, Jennifer Roche, Kristy Snyder, Ryan McCall

**Affiliations:** Department of Psychology, University of Memphis, Memphis, Tennessee, United States of America; Indiana University, United States of America

## Abstract

Much evidence exists supporting a richer interaction between cognition and action than commonly assumed. Such findings demonstrate that short-timescale processes, such as motor execution, may relate in systematic ways to longer-timescale cognitive processes, such as learning. We further substantiate one direction of this interaction: the flow of cognition into action systems. Two experiments explored match-to-sample paired-associate learning, in which participants learned randomized pairs of unfamiliar symbols. During the experiments, their hand movements were continuously tracked using the Nintendo Wiimote. Across learning, participant arm movements are initiated and completed more quickly, exhibit lower fluctuation, and exert more perturbation on the Wiimote during the button press. A second experiment demonstrated that action dynamics index novel learning scenarios, and not simply acclimatization to the Wiimote interface. Results support a graded and systematic covariation between cognition and action, and recommend ways in which this theoretical perspective may contribute to applied learning contexts.

## Introduction

It seems natural to wonder how subtle movements of the body relate to the processes of the mind. Whether in analysis of “body language” in the mass media [1], or in carefully controlled experiments on detecting emotion and deception [2], it is for both practicality and curiosity that the dynamics of movement are studied during the unfolding dynamics of thought. In the past 20 years, cognitive scientists have subjected this topic to intense experimental scrutiny. Without exception it is found that the dynamics of bodily movements relate richly to the underlying processing that gives way to these movements. For example, the continuous movements of the hand have been shown to co-vary with underlying cognitive processes such as spoken-word recognition and categorization [3], [4], force and velocity of button presses co-vary with stimulus frequency and reward in lexical decision and simple reaction time tasks [5], [6], and even the rapid trajectories of the eyes' saccades can display subtle curvature depending on stimulus context during attention [7], [8].

Several lines of research combine with these findings of graded covariation to recommend that action and cognition be given more focus as joint interactive processes. For example, some computational models of action planning and production have accounted for wide ranges of data by assuming that these processes unfold continuously together [9], [10]. Extensive work by David Rosenbaum and colleagues has shown that motor planning and control can be partly understood through concepts drawn from perception and cognition [11]–[14]. In addition, a number of studies have shown that bodily contexts can feedback onto cognitive processes. For example, induced eye movements can improve problem solving [15], and the way the arm is used to generate responses can modulate how stimuli are subsequently processed [16]. These and numerous other findings suggest that cognitive and action systems may more richly interact than commonly assumed (a modest canvassing of this evidence could include Refs. 17–28).

Many researchers look to these data for novel insights into the cognitive system. One intuitive interpretation is that the flow of mental processing is continuous and dynamic [29], [30]. Because the cognitive system does not collapse a discrete decision onto brittle, simplistic movements, it can be argued that the processes leading into this decision are themselves continuous and graded, leading to this dynamic covariation of executed actions. Despite this approach, the idea that low-level details of actions may relate richly to longer timescale processes has been explored in other theoretical contexts, prominent among them the computational framework of ACT-R [31]. Whatever one's choice of theoretical banner, this exploration of cognition and action addresses a fundamental challenge facing the cognitive sciences: to bridge the various levels of complexity relevant to human brain and behavior. In this context, an outstanding puzzle is further elaborating the systematic relation between low-level, short-timescale characteristics of movement and high-level, longer-timescale processes, such as learning.

In this paper, we contribute to building this bridge through relating the dynamics of action and a relatively simple learning task. Anderson [31] provides a strong basis for this approach, and describes a research program that spans “seven orders of magnitude.” This is accomplished by showing that characteristics of cognitive processing (spanning tens of milliseconds) may be directly related to meaningful learning experiences (extending over hours)–thus spanning enough of Allen Newell's famous timescales [32] to achieve seven orders of magnitude. Anderson notes that this bridging, from cognitive processing into educationally relevant learning, is an important current obstacle in cognitive science. In related recent research, others have focused on action sequences in learning tasks (e.g., eye movements), how they may relate to successes in learning, and how an understanding of this relationship will permit computer adaptation and improvement of educational design [33]–[35]. In a review of this budding literature, Anderson observes that subsymbolic aspects of processing–the internal cognitive characteristics of individual decisions and actions–may relate directly to outcomes in extended learning experiences, but that further research is required to demonstrate this. The current work aims to contribute to this demonstration, and shows that the acquisition of paired associates can be indexed by the characteristics of unfolding action–its temporal extent, complexity, and force.

There has been extensive work in the area of motor control on the change evident in movements across training [36], [37]. In most of this research, the task is to some extent equated with the action patterns themselves. For example, it has recently been shown that eye and hand movements mirror each other in the learning of a challenging motor task (i.e., the eyes simulate arm movement) [38]. In this case, the learning *is* the patterns of motor movements acquired by participants. In the task employed here, we embed paired-associate learning in a computer interface that continually extracts aspects of their arm movements during training. Thus the task does not inherently involve the motor dynamics to accomplish it–the participants are learning symbol pairs, not movements. Unbeknownst to them, however, we continuously track the movements of the hand that occur on each trial. The characteristics of this dynamic behavioral signal relate directly to the learning that is taking place over many minutes. Results contribute to the bridging of levels that is hoped for [31], and further substantiates a fine-grained flow from cognition into action.

In what follows, we present 2 experiments showing the modulation of action dynamics across learning. Experiment 1 was conducted to demonstrate that latency, motion time, motion fluctuation, acceleration, and button-induced perturbation of a Nintendo Wiimote pointing device all change across learning of random symbol pairs. They also to some extent index correct and incorrect trials, showing that an individual's knowledge may be marked by the dynamics of their arm movement. A more direct evaluation of novel learning was conducted in Experiment 2, demonstrating that action dynamic measures reflect novel scenarios in learning, robustly indexing lack of knowledge in participants. We end with a discussion of the theoretical and practical implications of these results.

## Methods

### Experiment 1

#### Participants

Participants included 21 (19 females, mean age 21.2) University of Memphis undergraduate student volunteers from the psychology participant pool that self-reported having corrected or no visual impairments. Each undergraduate received partial credit toward meeting his or her Introduction to Psychology course research participation requirement. Procedures for this study were approved by the University of Memphis Committee on the Protection of Human Research Participants. Before the experiment, participants provided written informed consent consistent with guidelines specified by that committee.

#### Interface display and device

Participants stood behind a small, 76 cm high table on which an Epson LCD projector was placed. This projected an Apple Mac mini's display along the long part of an oblong laboratory room and onto the wall at the end of the room (3.8 m×1.8 m). The projection screen was approximately 1.4 m in width (29.1° visual angle), and participant position was approximately 2.7 m away from it.

**Figure 1:**
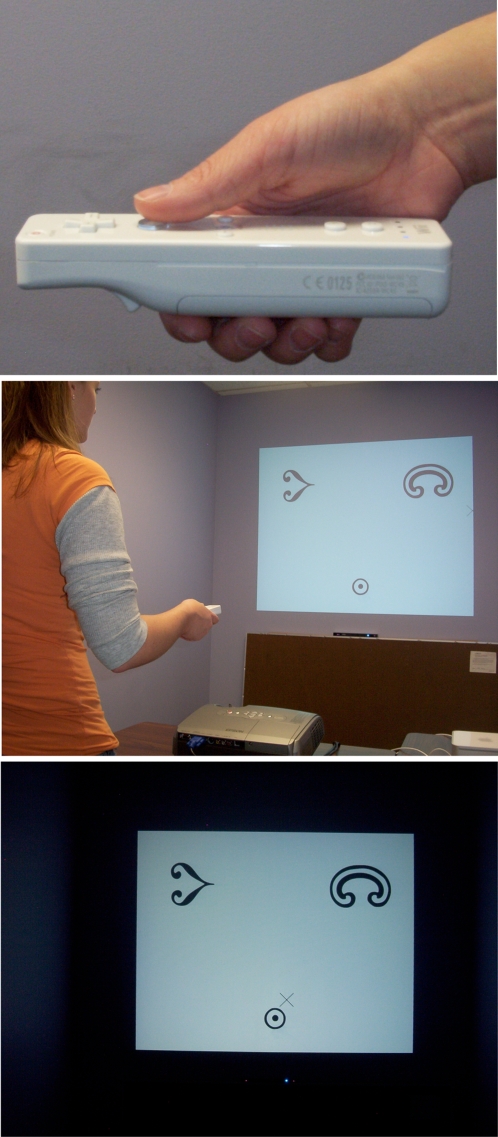
The experimental display and interface. Top panel: The Wiimote is held in the dominant hand, with the thumb engaging the remote's A button to click in the experimental software. Middle panel: A view of the overall context, with the light in the room on. The arm is held above the projector, and the remote controls a cursor that selects the correct match. In this image, the trial initiation target at the bottom center is present. Bottom panel: Participants performed the learning task with the lights dimmed.

In this visual context, we sought an affordable technique to extract rich action dynamics data. The Nintendo Wiimote (Figure 1, top panel) can be used as a wireless, arm-extended pointing device by having it communicate with a computer equipped with the Bluetooth transfer protocol. A Macintosh framework called DarwiinRemote (© 2006, Hiroaki Kimura) accomplishes this interfacing. See Text S2 for detail on the source code modification by this paper's authors, and links to further development of the software by other programmers. Because the Wiimote is equipped with three accelerometers, one for each axis of three-dimensional movement, we modified the source code of DarwiinRemote to store these axial acceleration data (sampled at approximately 90Hz) in a data file that could be synced with the experimental presentation software (sampling cursor data at approximately 80Hz). At the base of the projection screen was a Nyko infrared emitter. Like the video game console's sensor, this is to provide the Wiimote a frame of reference for computing position (see Text S1).

Participants stood behind the small table, with the Wiimote in the dominant hand extended out (Figure 1, middle panel). The height of the table permitted all participants to have their arm comfortably located above the projector, approximately above the lens from which the computer's display was projected onto the wall. Using this immersive context (lights were dimmed during the experiment; Figure 1, bottom panel), both x,y-pixel coordinates, and x,y,z-axial acceleration data were sampled throughout the experiment.

#### Materials and procedures

This experiment used a match-to-sample design for training paired associates of unfamiliar symbols. One shape was designated as the pair's sample, the other the pair's match/comparison stimulus. Trials began by first displaying the correct match and a random incorrect match at either the top left or right position, determined randomly, with a separation of approximately 58cm or 12.3° visual angle. Participants then clicked a trial initiation circle at the bottom center of the screen. At this location (after their Wiimote click) the sample shape appeared. Participants then moved the Wiimote-controlled cursor to the upper left or right to click on an answer. Feedback was provided in the form of a green “correct” or red “incorrect” in text in the space between the sample and matches at about the center of the projected screen. Participants saw 150 learning trials, and across training saw each pair at least 9 times and at most 11 times, with most seen 10 times evenly distributed across training. Notably, the task was designed to elicit guessing among the first few trials. Therefore, participants were informed of the possibility of guessing until they learned the matched pairs.

Experiment 1 included a total of thirty different symbols taken from the Bodoni Ornament font set (for examples see Figure 1, middle and bottom panels). This font was chosen because the shapes were not easily namable, but had a variety of overlapping features (symmetry, radiality; see Figure S1). Images occupied approximately 23 cm or 4.9° visual angle on the projection screen. At the beginning of the experiment, the experimental software randomly combined these symbols to provide fifteen pairs. Thus, each participant saw a completely different set of randomly established symbol pairs. On any given trial, one of the fifteen pairs was randomly presented to the participant.

Both the researcher and experimental presentation software provided instruction to the participants to ensure that they understood the task. Python (python.org) and the Pygame (pygame.org) video game module were used to present training trials and sample the Wiimote-controlled cursor movements as streaming x-y coordinates. Participants largely reported enjoying the task because it resembled a game. Participation on the task required no more than 15 minutes.

#### Measures

A methodological contribution of this paper is adapting the Nintendo Wiimote for behavioral experimentation. Among many interesting characteristics of the Wiimote as a pointer device, one particularly relevant for this experiment is that the arm is held out and will exhibit natural sway during the task. In computer-mouse contexts, the fixed two-dimensional surface of a table acts as a stabilizer granting a clearer “latency” before executed movement. The Wiimote does not have this stabilizing contribution from a table, and we therefore defined an “escape region” of a certain pixel distance from the point of origin, where a trial was initiated. When the cursor departs this escape region, we can separate the first portion of the Wiimote trajectory as the “latency,” and the remainder of the trajectory as the in-motion component executing the decision. This escape region changes over learning, with extensive sway early in the experiment (generating a broader escape region) and more quickly executed movements later (generating a more narrow region).

**Figure 2:**
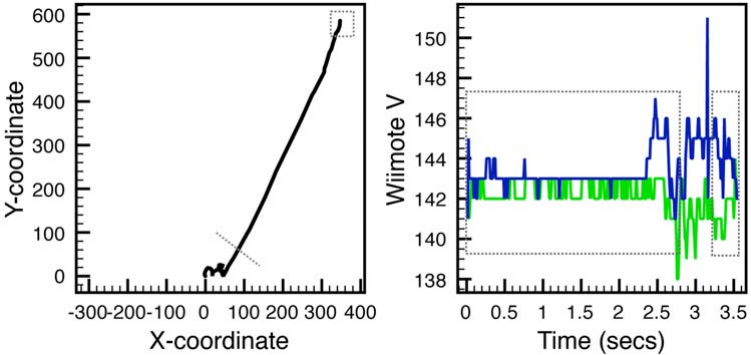
Visualization of extractable Wiimote data. Left panel: An example Wiimote trajectory from the bottom center to the right match. The trajectory demonstrates the sway present in the arm prior to committing to the movement, during latency. When the arm moves outside the 100-pixel escape region (dotted line), this provides the in-motion segment of the trajectory. The small dotted square is the location at which a button press is initiated. Right panel: The x- (green) and y-axis (blue) accelerometer data for that same trial. During latency, a resting voltage signal is present (the at-rest voltage generated by the accelerometers). This voltage is modulated (up or down, depending on direction of movement) when the Wiimote is displaced, shown between the two dotted boxes. This example trial has a brief in-motion segment. Acceleration range was computed by subtracting the mean latency acceleration from the absolute maximum acceleration during motion. The rightmost dotted box is the region in which perturbation of the remote is inspected for voltage range.

After inspecting a random set of participant data plots of individual trials, it was found that most latency sway occurred within a 25- to 100-pixel radius around the origin. Because the focus of these experiments is exploring the information afforded by the dynamics of action itself, all dependent measures were based on a conservative escape region of 100 pixels around the trial initiation click (15 cm, 3.2° visual angle). This separates a Wiimote trajectory into two components, one for “latency” during which the cursor remains within a 100-pixel radius, and another for the motion time to final click, after the escape occurs (see Figure 2, left panel). We expect that latency and motion time both diminish as participants acquire knowledge of the symbol pairs.

In addition to these two dependent measures of the relative time of decision and movement, we explored a measure of complexity of the movement component. Previous work has used sample entropy [4], [39], and though grounded on applications in the natural sciences, we chose a measure that provides a more intuitive interpretation. We calculated, along the axis of decision (x-axis), how many reversals of direction were present in the movement component of the trajectory. This gives a count of the “x-flips,” a directly interpretable quantity representing the complexity of movement in the decision axis. When the two possible matches are not easily decided between, they may act to draw the hand towards them early in learning [39]. If this is so, we should find more x-flips during early parts of learning, and then a diminishing across learning. We counted any change in direction as such a flip, permitting us to capture any low-level fluctuation occurring in the Wiimote movement.

The Wiimote's three accelerometers generate a voltage signal for the three axes of movement (see Text S2). The accelerometer data are digitized voltage “points” around a baseline voltage present when the Wiimote is at rest. We chose three accelerometer measures to supplement the cursor analysis. All three were based on the axes relevant to the task (x- and y-axes). For the first two, we used the maximum acceleration generated during movement along both the x- and y-axis of movement (see Figure 2, right panel). To do this, we calculated the mean accelerometer signal during the latency portion of a trial, and extracted the absolute maximum difference in the voltage signal after this (during motion) with respect to this mean latency voltage by subtraction. The final 100ms of motion were removed from the data of each trial due to the potential influence of the button-click perturbation on the accelerometers. When the remote is clicked, it generates a weak perturbation in these voltage signals proportional to how hard the participant presses the Wiimote button. As a final measure, we tracked this by extracting the range of voltage generated 100 ms prior to and 200 ms following the button click. This “perturbation” of the Wiimote accelerometers was calculated as the mean range of voltage signal of x- and y-axes during that final portion of a trajectory (see Figure 2, right panel).

In summary, we employed a range of dependent measures drawn from the Wiimote's movement and accelerometers. We anticipate movements exhibiting more “confidence” as learning becomes more robust: faster and simpler movements. The accelerometers may reveal that movements come to carry a higher acceleration along the direction of decision (x-axis). In addition, as the participants become more confident in responding, the perturbation on the Wiimote seems likely to increase. Overall, these measures serve as an array of potential dynamic “signatures.” These dynamic bodily characteristics of responding were predicted to index the progress of the longer-timescale cognitive task of paired-associate learning.

### Experiment 2

The set up for Experiment 2 was almost identical to Experiment 1. The only difference is the order of presentation of the pairs. We separated the 15 pairs into 3 blocks of 5 learning domains. These were presented separately in blocks of 50 trials (for 150 blocks in total). We can therefore be certain that at the onset of a new block, participants are merely guessing. 50-trial blocks permitted 2 additional junctures in the experiment at which this should be observed. Like Experiment 1, each pair was seen at least 9 times, at most 11, and most seen 10 times within each block. We extracted the same dependent measures here, and explored their modulation at the onset of the novel pairs.

#### Participants

Participants included 25 (21 female, mean age 19.4) University of Memphis undergraduate student volunteers from the psychology participant pool that self-reported having corrected or no visual impairments. Each undergraduate received partial credit toward meeting his or her Introduction to Psychology course research participation requirement.

#### Interface display and device

The same display setup and Wiimote device from Experiment 1 were used.

#### Materials and Procedures

The same 30 images from Experiment 1 were used here. Again, within each participant, these were combined randomly so that 15 pairs served as the domain of learning.

In Experiment 2, rather than presenting all 15 pairs across training, we divided the stimulus set into 3 blocks of 5 pairs. For the first 50 trials of the experiment, only 5 pairs were shown. The incorrect match used in these trials came from the other possible matches in those 5 pairs. Participants were thus only exposed to a smaller domain of 5 pairs, 10 shapes. At trial 51, the domain of learning became the second block of 5 pairs. Participants will have acclimated to the Wiimote device, and have robustly learned the 5 prior pairs, but will now be completely unfamiliar with the new 5 pairs to be presented for the second block. Participants were not told of this. A third block of 5 was presented for the final 50 trials.

#### Measures

The measures described in Experiment 1 were also extracted here.

## Results

### Experiment 1

#### Outliers and means

There were several extremely long learning trials, with a few almost 20 seconds in length. Because the following regression analyses used pooled data across trials, extreme outliers could substantially influence our results. We thus removed any trials that were more than 3 standard deviations (approximately 7 seconds) from the overall mean trial length. This amounted to less than 2% of the data. Interestingly, as would be predicted, these 61 trials (of 3150) were distributed mostly in the first half of the experiment (50 vs. 11, χ(1) = 24.9, *p*<.001) as learning is starting. This does provide a first piece of evidence suggesting that more extensive movements occur with lack of knowledge. This also ensures that the following analyses are sufficiently conservative, and not due to these few extreme trials. Overall means are presented in Table 1.

**Table 1:** Correlations in Experiment 1 between trial number and mean measures (across participants)

Variable	M (SD)	*r*_variable, trial number_
performance	71% (46)	.75**
latency	716.9 ms (561.5)	−.80**
motion time	1290.8 ms (1010.6)	−.45**
x-flips	3.4 flips (4.2)	−.32**
x-accel. range	5.2 (3.4)	.34**
y-accel. range	5.2 (3.1)	.30**
perturbation	4.7 (2.5)	.61**

** p<.01

#### Cursor analysis

**Figure 3:**
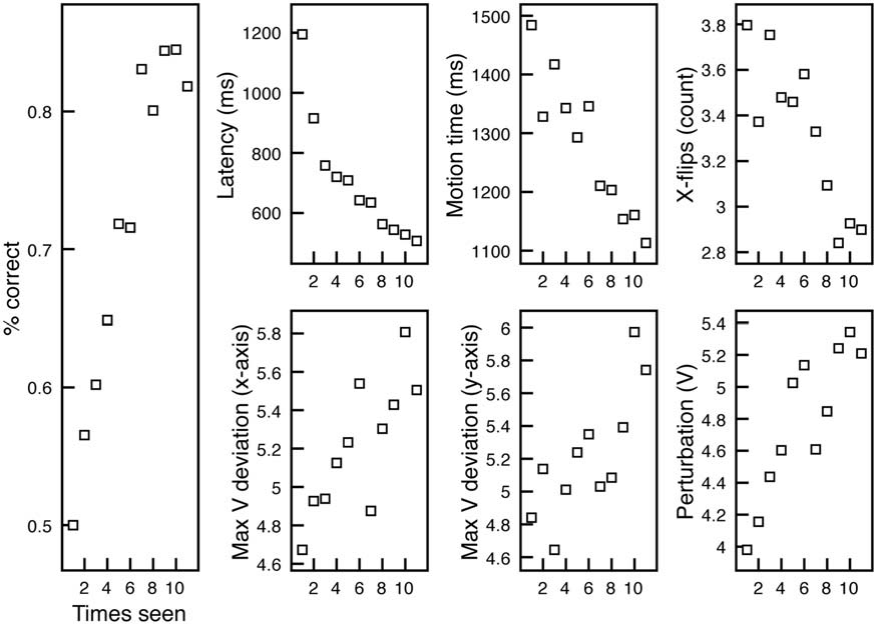
Results of Experiment 1, with means computed over presentation order for each symbol pair. All cursor measures diminish across symbol presentation order, while acceleration measures rise.

In a first analysis, the general trend across learning was judged by performing simple bivariate correlations with mean measures pooled within each trial number (1-150) across participants. All three variables exhibited a significant drop across learning (see Table 1). Because item analyses are not possible (pairs are fully randomized for each participant), we conducted an additional analysis of how measures change each time a particular pair is seen. This captures how measures change for each exposure of a symbol pair. We thus pooled not across trials of the experiment, but across the ordinality of presentation for pairs: The number of times a pair is seen, from presentation 1, to a possible 11. Like the trial-based correlations, all trends are significant for each measure (*p*'s<.001), indicating that measures significantly change across pair presentations. This pooling of data provides a more orderly presentation of results, and we use presentation order in Figure 3 to show data trends.

To assess whether incorrect trials were marked by different action dynamics, we conducted an additional analysis using a sequential regression model for each dependent measure. We separated correct and incorrect trials across participants, and for each of 150 trial numbers computed mean measures. In the first step of the models, we included two predictors: the trial number (1-150) and a variable coding whether a trial was correct (1) or incorrect (0). In a second step, we added an interaction term composed of the product of the predictors (as described in Ref. 40). If the dynamics mark knowledge, one would predict that the diminishing of action dynamics over correct trials should be more substantial compared to incorrect trials, for which there was likely more uncertainty. This should produce a significant interaction, judged by the reliability of the change in *R^2^* when the interaction term is added into the model.

The first step in the models for all three measures was significant, accounting for 38%, 8%, and 4% of latency, motion time, and x-flips, respectively (*p*'s<.005). The interaction term was only reliable for motion time, with added variance of 2% explained (*p*<.05). Less than 1% of latency was marginally accounted for by the interaction (*p* = .052). In the final model with all predictors, only motion time and x-flips were significantly predicted by the correct/incorrect status of the trials (see Table 2). This indicates that there could be diminishing of the dynamics over time, with parallel changes across trials but at a different level depending on the performance on a trial (indicated by the negative standardized coefficient–in a correct trial, the dependent measures are predicted to decrease by at least 13% of a standard deviation). So, while the interaction terms were very weak, the still-significant correct/incorrect predictor indicates that the action dynamics significantly relate to performance.

**Table 2:** Sequential regression results with added product term in Experiment 1 predicting mean measures (across participants)

Variable	*β*_correct/incorrect_	*β*_trial number_	*β*_trial×correct/incorrect_	*ΔR^2^*
latency	.02	−.53***	−.13†	.01†
motion time	−.22***	−.04	−.19*	.02*
x-flips	−.13*	−.08	−.11	.01
x-accel. range	−.04	.22**	−.03	.00
y-accel. range	.02	.08	.08	.00
perturbation	.10†	.27**	.10	.01

* p<.05

** p<.01

*** p<.001,

† <.10

**Figure 4:**
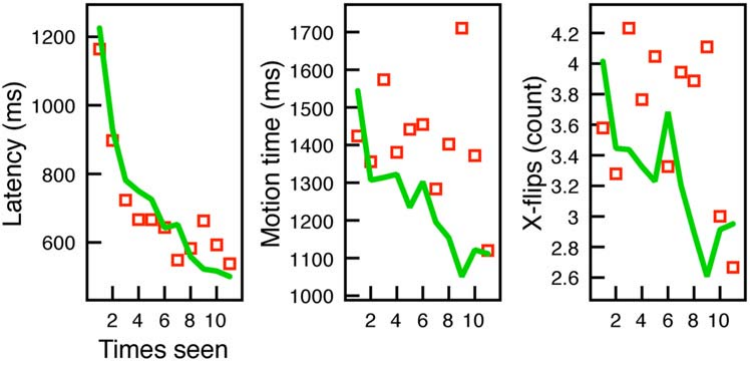
Presentation of the interaction between correct (green line) and incorrect (red points) trials and presentation order. Latency does not seem to index correct/incorrect trials across presentation order for each pair. However, in-motion time and x-flips drop relatively more for correct trials compared to incorrect trials, indicated by the significant interaction term (see text for details).

In the same follow-up analysis based on the pair presentation order, all first steps of the model were again significant (*p*'s<.05). No measure was significantly predicted by the interaction term, while again both motion time and x-flips were significantly explained by the trial performance in the final step of the model (*p*'s<.05). As in the trial-based regressions, only the post-latency measures related to actual performance. Figure 4 presents these measures across presentations for correct and incorrect trials separately.

#### Accelerometer analysis

Due to irregularities in some Wiimote tracks, 3 participants had their Wiimote data excluded from this analysis. It was discovered later that this was due to low battery power in the Wiimote and the infrared emitter. Data from the remaining 18 participants served as the basis for this analysis. Outlier trials from the previous analysis were also excluded from this analysis. These same trials consisted of less than 2% of the data from the remaining trials. In addition, due to complexities in pairing cursor-tracking data with Wiimote accelerometer data, an additional set of trials had to be discarded (see Text S2). In all, less than 4% of the trials across the 18 remaining participants were lost.

In the simple bivariate results, all three measures exhibited significant rise over time, indicating that participants on average are generating larger ranges of acceleration and Wiimote perturbation during learning (see Table 1). The same regression analysis as in the previous analysis was used here, with correct/incorrect trials coded separately along with the predictor of trial number. The first step of this analysis was significant for perturbation and x-acceleration range (12% and 4%, respectively, *p*'s<.005), while y-acceleration range was only marginally explained at 2% (*p* = .06). In the second step (with the interaction term added), it was found that not one of the accelerometer-based measures was significantly predicted by it (see Table 2). However, perturbation was still accounted for by trial number and marginally so by correct/incorrect status of a trial. In the follow-up presentation order analyses all three dependent measures had significant first steps (*p*'s<.01). None was accounted for by performance or the interaction term, however.

#### Summary

Action dynamics require less time to initiate, are faster to complete and exhibit less x-axis flipping, as learning unfolds. This appears not to be simply because participants are familiarizing themselves with the experimental interface. The significant correct/incorrect prediction of motion time and x-flips suggests that characteristics of arm movements mark whether a participant has knowledge of a pair. In addition, the accelerometer results show that arm movements achieve higher acceleration as learning unfolds, and seem to depress the Wiimote button generating higher perturbation in the accelerometers. Overall, the measures derived from movement component for the trials co-varied more systematically with learning performance. This is not to say that both latency and end-response measures are uninformative–only, at the very least, that action dynamics may importantly supplement these measures.

In general, the interaction term in these analyses was not a reliable predictor across measures. It is important to note that in early trials correct responses may have involved substantial guessing, making the use of just incorrect trials to test this interaction particularly conservative. In fact, in this first experiment, this is the only convenient means by which we might separate low vs. high level of familiarity with certain pairs across training. In Experiment 2, we created a scenario in which we can tell when participants would be guessing. This permits a more direct test of knowledge level and whether the dynamics of the arm can serve as indices of this.

### Experiment 2

#### Outliers and means

We used the same criterion for outliers in this analysis as in Experiment 1 (3 standard deviations, trials approximately 7 seconds or greater). The resulting data discarded amounted to less than 2% of all trials (65 of 3750). Means are presented in Table 3, and are similar to those of Experiment 1. This time, we inspected how these trials were distributed in the 3 blocks, as we did for first half/second half test in Experiment 1. Most discarded trials did occur in the first block, indicating that participants may have been partly acclimating to the task and interface (32 vs. 15 vs. 18, χ(2) = 7.6, *p*<.05). However, the effect is considerably smaller, with more occurring in the two later blocks of just 50 trials, compared to the 11 in the 75-trial second half of Experiment 1. The following analyses test whether the introduction of novel pairs does indeed modulate the action dynamics of the learner.

**Table 3:** Correlations and sequential regression results in Experiment 2 predicting mean measures (across participants)

Variable	M (SD)	*r*_variable, trial number_	*R*	*β*_trial number_	*β*_trial in block_
performance	80% (40)	.30**	.67***	.1	.6***
latency	693.0 ms (597.0)	−.68**	.78***	−.55***	−.40***
motion time	1291.2 ms (998.4)	−.33**	.40***	−.25**	−.25**
x-flips	3.4 flips (4.1)	−.12	.20†	−.07	−.16†
x-accel. range	5.3 (3.6)	.34**	.35***	.30***	.10
y-accel. range	4.6 (3.3)	.09	.09	.09	−.01
perturbation	4.4 (1.8)	.63**	.67***	.55***	.23**

** p<.01

*** p<.001,

† <.10

#### Cursor analysis

As in Experiment 1, a first analysis of the general trends across learning used bivariate correlations, presented in Table 3. All variables except x-flips exhibited a significant drop across learning.

One would expect weaker results on these simple correlations given the interruption by new material at two points during training. In fact, it seems surprising that these values do exhibit a significant relationship with trial number. It is possible that participants are not modulating in the context of novel items, but simply find the task harder and take more time to acclimate to the device. To test this, we ran a simultaneous regression model for each measure, entering both trial number (1-150) and corresponding block trial number (0-49). For example, for the first trial of the second block would have an overall trial number of 51, but a block trial number of 0. If participants are merely taking longer to adjust to the difficulty, then controlling for overall trial number, the block trial should explain no variance in the measures.

**Figure 5:**
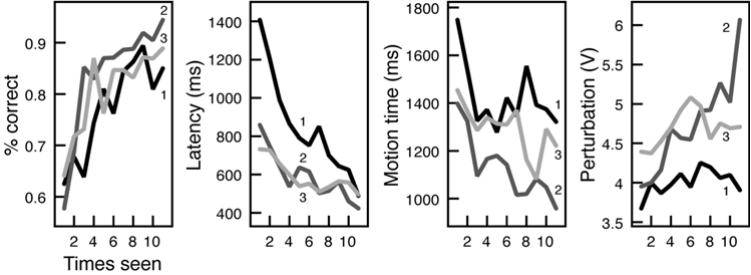
Results of Experiment 2 presented by presentation order for each pair. Each block is presented as a separated line, labeled using block number and shaded from dark (first block) to lighter (third block) lines.

Table 3 shows the standardized coefficients in these models. Latency and motion time both have significant models, accounting for 60% and 16%, respectively (*p*'s<.001). The measure of x-flips only attained marginal significance, with 4% of its variance explained by trial and block trial (*p* = .055). The individual coefficients for trial number and block trial number are significant in models of both latency (*β*  = −.55 and −.40, respectively, *t*'s>7.0, *p*'s<.001) and motion time (−.25 and −.25, respectively, *t*'s>3.1, *p*'s<.005). It seems that overall training progress does predict a general diminishing of these measures, but at the point of a novel block, there is a significant modulation of them as well. In a follow-up presentation-order analysis as in Experiment 1, results are overall consistent. Overall presentation order and within-block order are significant for latency and motion time (*p*'s<.05). The x-flip measure was not significantly accounted for by presentation order. Means of this analysis are presented in Figure 5.

We were surprised that the measure of complexity, x-flips, did not show reliable modulation in this second experiment. In follow-up analyses, we explored whether x-flips in the latency portion of the Wiimote trajectory showed modulation over time. As described above, the Wiimote lacks the stabilizing surface of a table, and so will reveal natural sway in the arm during trials. We thus extracted these first (latency) trajectory portions in Experiment 2, and conducted similar regression analyses. The x-flips in the pre-escape region during “latency” exhibited a strong trend over trials, but also within trial block number (*β*'s = −.52 and −.33, respectively *p*'s<.001). In the presentation order follow-up analysis, these coefficients are also significant (*p*'s<.001).

The measure of x-flips affords an intuitive measure of complexity, but only when the complexity consists in non-monotonic fluctuation in the x-axis movement. In other words, if subjects produced fluctuations that are monotonic along the x-axis (not changing direction) this x-flip measure will come up empty. Entropy-based measures are available that may serve to quantify this, and we used sample entropy [41] that measures the relative “disorder” of a time series. As in previous work [4], [39], we interpolated all trajectories to 101 time steps, and computed x-axis change (Δx = x_t+1_-x_t_). The time series is therefore representative of the *shape* of x-axis fluctuation, and is a consistent length for each trajectory. Sample entropy is then computed on these interpolated x-axis fluctuations by counting the number of x-axis fluctuation (Δx) sequences of length 3 (M_3_) that stay within a given tolerance (here, SD of Δx), then counting how many sequences are retained when the window size is extended to 4; thus sample entropy = -ln (M_4_/M_3_) [41].

**Figure 6:**
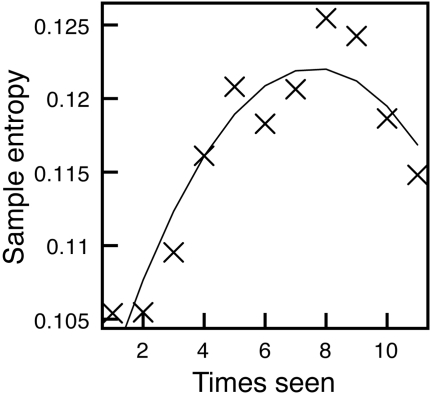
Sample entropy for each presentation order collapsed across blocks. Values initially rise then drop near final presentation orders.

In a similar multiple regression model with trial and block trial as predictors, entropy is significantly predicted by both (*p*'s<.001). Standardized coefficients for trial and block trial, .47 and .23, respectively, suggest that complexity is increasing as learning unfolds. However, when mapping sample entropy on presentation order as in previous measures (shown in Figure 6) there is a distinct nonlinearity visible. Entropy rises, but then drops at the tail end of the blocks. A squared term for within-block order and trial is significant in both analyses (*p*'s<.05). In fact, in the follow-up presentation order analysis, only overall presentation and squared within-block order terms were significant, suggesting that within a block the nonlinearity best characterizes the complexity of the x-axis fluctuations. This indicates that, while the reversals of direction (x-flips) may generally decrease to some extent, the underlying complexity of the signal measured through sample entropy displays a within-block nonlinearity. As we discuss below, some recent work on problem solving predicts this [42], [43].

As a final check of our overall simultaneous multiple regression strategy, we included block trial number in a simultaneous model for the data from Experiment 1. Table 4 shows these results, and as expected, there is no significant change given a corresponding block trial number in those data.

**Table 4:** Regression results using Experiment 1 data predicting mean measures (across participants)

Variable	*β*_trial number_	*β*_trial in block_
performance	.76***	−.03
latency	−.79***	−.03
motion time	−.43***	−.06
x-flips	−.30***	−.05
x-accel. range	.29**	.14†
y-accel. range	.27**	.10
perturbation	.60***	.05

** p<.01

*** p<.001,

† <.10

#### Accelerometer analysis

Again, due to irregularities in the Wiimote tracking, 5 of 25 participants had to be removed from the accelerometer analysis. 3 of these were due to the low-battery issue, and 2 due to large difficulties pairing the trials. Outlier trials consisted again of less than 2% of the remaining data, and lost trials due to Wiimote pairing less than 4%.

In bivariate analyses, x-acceleration range and perturbation both increased over training (see Table 3). The y-acceleration measure was not significantly predicted by any of our variables in any analysis. Using the same regression analysis as in the cursor analysis, including trial number and block trial number, perturbation and x-acceleration range were significantly accounted for by these predictors, with 45% and 12% explained, respectively (*p*'s<.001). For x-acceleration range, only the overall trial number significantly contributed to the model (*β* = .30, *t*(2) = 3.7, *p*<.001), though both overall trial number and block trial number contributed to the explanation of variance in perturbation (*β*  = .55 and .23, respectively, *t*'s>3.6, *p*'s<.001). Presentation-order analyses were exactly consistent with these patterns of significance. Using presentation order, mean perturbation and x-acceleration range measures are shown in Figure 5.

#### Summary

Except x-flips, all cursor-based measures showed changes at the onset of a novel learning. Of the accelerometer measures, only the perturbation measure showed a significant change at these junctures in the experiment. The x-acceleration range changed significantly across all 150 trials, but was not predicted significantly by the block number variable. This indicates that the participants are generating higher acceleration along the axis of decision as the experiment proceeds, but do not modulate this acceleration in the face of novel stimuli. They do, however, modulate the force of the Wiimote press.

The analysis using sample entropy also suggests an interesting nonlinearity occurring within blocks of this experiment. This result is in fact predicted by very recent work by Stephen, Dixon, and Isenhower [42], in which entropy-based measures index the progress of solving a gear problem (see also Ref. 43 for related discussion). These authors argue that the entropy signal reveals organizational change within a complex nonlinear dynamical system. As the system undergoes substantial reorganization, entropy should increase then undergo a subsequent drop as the system stabilizes into a new configuration. Sample entropy here may also be indicative of system change as described by these authors.

## Discussion

The goal of these experiments was a simple and intuitive one. We aimed to show that the dynamic characteristics of action reflect ongoing learning in a cognitive task. Both experiments revealed this. Experiment 1 showed that features of action dynamics grow more “confident” over a learning task, and can mark the performance of the participant, indicating whether or not they had acquired particular knowledge. Experiment 2 revealed that these characteristics generally index learning, not just motor familiarity with the device. When novel items are presented to participants in the flow of the experiment, there is a reliable modulation in these dynamics: How long it takes for the arm to move, how long the arm is in motion, and how much perturbation is placed on the pointing device.

There are a number of limitations of this study that future work should seek to improve. First, the paired-associate learning is extremely simple and requires little time to accomplish. More complex learning processes should be explored (e.g., learning content from text), perhaps better integrating the approach taken here with real-world learning tasks (see also below). Despite this, the fact that the learning task was extremely simple may have provided a more conservative context for establishing the covariation of action dynamics with cognitive processing. Despite the simplicity, the movements of the Wiimote modulate across the task, marking both performance and novel learning contexts. It seems equally plausible that more extended, challenging learning tasks could invoke even stronger action dynamics indices.

A second limitation is the kind of learning task explored. Multiple-choice learning contexts are by no means rare [44], but are not regarded as effective means of conveying novel information. Future work should adapt other learning tasks (e.g., hypertext learning environments, see Ref. 45) and embed action dynamics in these tasks to explore possible indices of learning and comprehension.

Finally, there is a need for theories that invoke cognition-action interaction to articulate in more detail when and when not particular action measures will co-vary with cognitive processing [46]. While we show that a self-organized dynamical systems approach predicts our finding of entropy change in Experiment 2 [42], there is still a need to pursue more detailed predictions of this sort across the kind of measures used here. One approach that will surely contribute to this question is further articulating the underlying neurophysiological systems that plan and produce action, and how relevant pathways from “decision” processes feed into them [19], [22], [24]. Another means of obtaining detailed prediction is to devise computational models that instantiate the proposed interaction [9], [10], [47], [48]. For example, Schutte and Spencer [10] have used a computational model based on dynamic field theory [9] to account for the modulation of action dynamics when moving across different spatial extents.

Despite these limitations, we draw methodological, theoretical, and applied implications from the current studies. The primary methodological contribution is adapting for the first time the Nintendo Wiimote pointer for use in basic behavioral experiments. Some details are supplied in supporting materials, and more work is needed to better integrate this device in experimental work. In fact, given the 3-dimensional accelerometer data, and the immersive context in which this pointer device can be used, a variety of novel contexts for behavioral experiments may be devised. One particular benefit of the Wiimote is the instability present in the arm during pre-decision processes. This may permit detailed analysis of the subtle movements during that unfolding decisions process as a potential additional index of learning and comprehension. The additional x-flips analysis offered above attests to this.

As described in the Introduction, we draw theoretical implications from these data. They contribute to two broad goals in cognitive science. One is to show that cognition and action relate in systematic and rich ways. These data support this question, and provide further support to the deep repository of empirical evidence that the “effectors” do not exhibit simple linear productions from the “privileged” central processes [49]. Instead, the dynamics of action co-vary in systematic ways as cognitive processing is unfolding. Whether one's interpretation of these data rests on dynamical formalisms [29] or some other framework [31], it is clear that the flow of information from cognition into action is taking place in a way that requires a richer perspective on their interaction. Related to this, the second broad goal is to demonstrate how low timescale processes can relate to longer timescale processes during real-time cognitive processing. In these experiments, we have shown covariation between action patterns unfolding in 100's of milliseconds and learning that is taking place across many minutes.

Finally, the rich layering of timescales, and their potential interaction, suggest novel educational adaptations of these findings. The theoretical approach described above recommends finding ways to continuously track learners in a variety of more or less complex cognitive tasks. Whether learning to read or reason, the systematic flow of information from cognition into action shows that dynamic action variables may mark learning in ways that discrete performance measures may not have access to (e.g., see Ref. 50). These dynamic indices could supplement traditional performance measures to aid in educational technologies. In general, by paying attention to action, new avenues of discovery in both theoretical and applied contexts are possible [51], [52]. The results reported here substantiate this sentiment.

## Supporting Information

Figure S1 These are the 30 Bodoni symbols used in Experiments 1 and 2. For each participant, these symbols were randomly paired to form the paired-associates that the participants learned across 150 trials of training.
(4.25 MB TIF)

Text S1 (0.03 MB DOC)

Text S2 (0.03 MB DOC)
