# Supplementary figures and images for: Exploring Action Dynamics as an Index of Paired-Associate Learning
Source: PLoS One. 2008 Mar 5;3(3):e1728. doi: 10.1371/journal.pone.0001728 (PMC2253184; doi:10.1371/journal.pone.0001728)

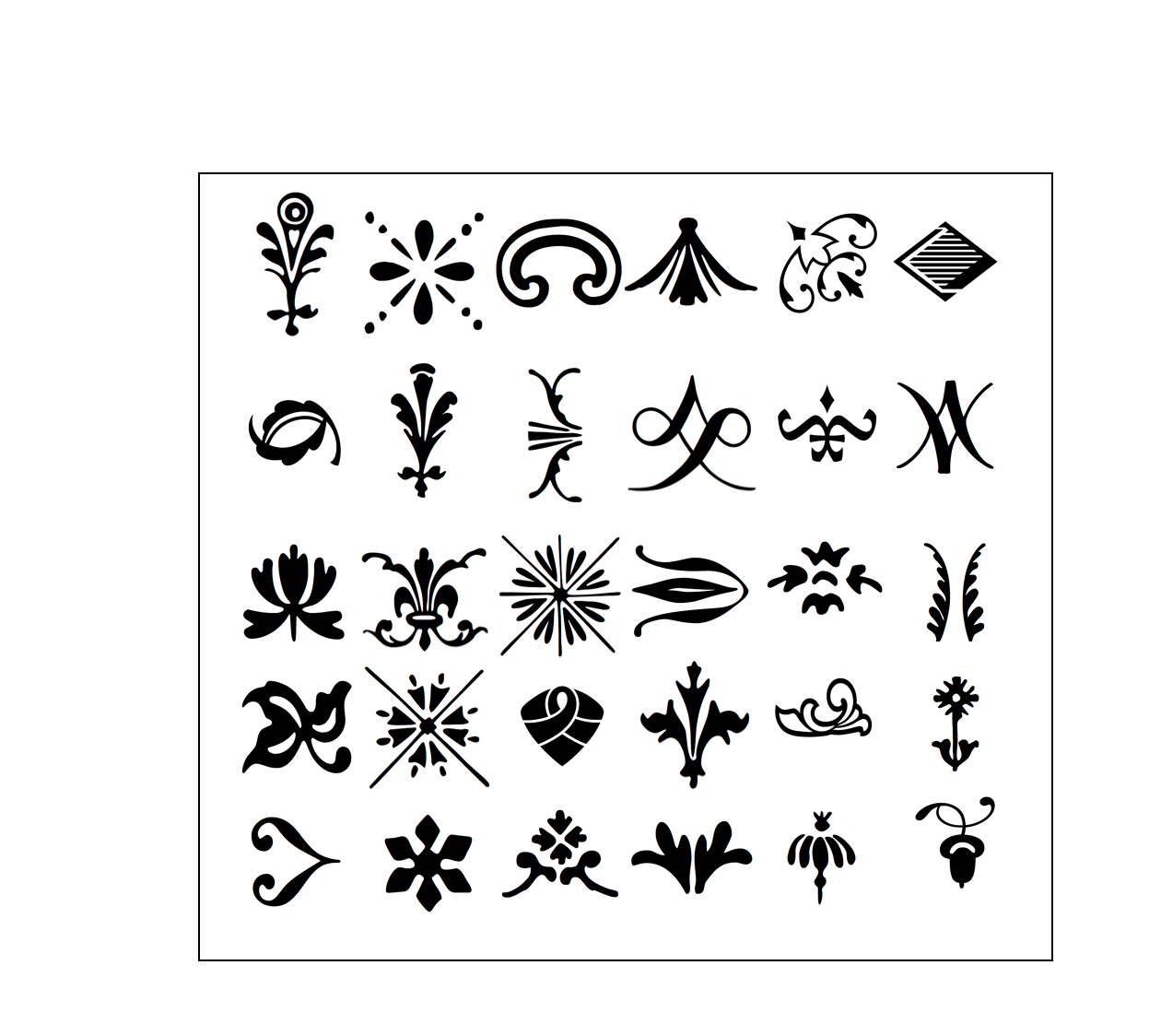

Supplement: Figure S1 — These are the 30 Bodoni symbols used in Experiments 1 and 2. For each participant, these symbols were randomly paired to form the paired-associates that the participants learned across 150 trials of training. (4.25 MB TIF) [file pone.0001728.s001.tif]
