# Supplementary material for: Exploring Action Dynamics as an Index of Paired-Associate Learning
Source: PLoS One. 2008 Mar 5;3(3):e1728. doi: 10.1371/journal.pone.0001728 (PMC2253184; doi:10.1371/journal.pone.0001728)
Supplement: Text S1 — (0.03 MB DOC) [file pone.0001728.s002.doc]

## Exploring Action Dynamics as an Index of Paired-Associate Learning

# Supporting Information

Rick Dale, Jennifer Roche, Kristy Snyder, and Ryan McCall

Department of Psychology

University of Memphis

Memphis, TN, 38152

**Corresponding author:**

Rick Dale

Email: radale@memphis.edu

Phone: (901) 678-4938

Web: http://cia.psyc.memphis.edu/rad/

## Text S1: Hardware

Besides a computer, there are three (two crucial and one optional) hardware components needed to setup the Nintendo Wiimote as a peripheral device for experimental use. The two key components include: the Wiimote itself and a wireless infrared (IR) emitter. The optional part, used in these experiments, is an LCD projector (a standard computer monitor will suffice).

### Nintendo Wiimote

Most electronics and department stores sell the Nintendo Wiimote for approximately $40 (US). It requires two AA batteries.

### IR emitter

We made use of the Nyko Wireless battery-powered (4-AA batteries) IR emitter. Many videogame stores sell these IR emitters, $20 (US), as extenders for the standard sensor bar that accompanies the Nintendo console so that game play can take place on a large-screen or project (to which the standard wires of the sensor cannot reach).

### Optional: LCD projector

We used an Epson LCD projector to provide participants with a large immersive visual context. This allowed participants to stand up during the learning process, and by maximizing the sensitivity of the DarwiinRemote software (see below), it allowed broader movements of the hand and arm to control the cursor. This maximized the kind of “immersive” sense this experiment provided for the participants, who mostly considered their participation fun, and “like a game.”

The details to accomplish this are described in numerous places across the Internet. An authoritative source regarding these issues can be found at the following reference:

[1] GNU/LINUX port for the Nintendo Wii by Wiili.org: Wiimote. (2007, October 29)  Retrieved December 3, 2007, from:

<http://www.wiili.org/index.php/Wiimote>
