# Supplementary material for: Exploring Action Dynamics as an Index of Paired-Associate Learning
Source: PLoS One. 2008 Mar 5;3(3):e1728. doi: 10.1371/journal.pone.0001728 (PMC2253184; doi:10.1371/journal.pone.0001728)
Supplement: Text S2 — (0.03 MB DOC) [file pone.0001728.s003.doc]

## Exploring Action Dynamics as an Index of Paired-Associate Learning

# Supporting Information

Rick Dale, Jennifer Roche, Kristy Snyder, and Ryan McCall

Department of Psychology

University of Memphis

Memphis, TN, 38152

**Corresponding author:**

Rick Dale

Email: radale@memphis.edu

Phone: (901) 678-4938

Web: http://cia.psyc.memphis.edu/rad/

## Text S2: Software and Issues

### DarwiinRemote

The final requisite component for integrating the Wiimote with the personal computer is a piece of software that handles the Bluetooth protocol to communicate with the Wiimote. We used an Apple Mac mini (using OS X 10.4.X), for which an open source software package called DarwiinRemote is available (our version was © 2006, Hiroaki Kimura; a new update has now been supplied: © 2007, DarwiinRemote Team, located here: <http://sourceforge.net/projects/darwiin-remote/>).

### Modification by the authors

We downloaded the source code to this project (written in Objective-C, for Apple Xcode) and modified it slightly to produce a datastream to a text file. This saves the x,y-coordinates computed by the Wiimote, and the 3D accelerometer data. These voltage signals are coarse and have a “resting” value when the Wiimote is not in motion (see Ref. 1, Text S1, for more detail). In order to track the Wiimote’s data, a pairing of the datastream from DarwiinRemote and the Python/pygame data (which stores experimental data) had to be conducted by hand using Matlab. This was done by graphing the Wiimote and Python x,y coordinates and matching them by hand using a Matlab-designed graphical user interface.

Future modifications to DarwiinRemote would usefully incorporate a way of communicating with the software through standard AppleScript means so that the status of the experiment can be stored with the rich source of Wiimote data.

### Issues

Lag: There is a resultant lag in the Python millisecond timing. Under multiple tests, this was empirically determined to be ~1.65 proportion in inflation of raw millisecond recordings. This scaling was applied to the mean data reported in the paper.

Batteries: Low batteries can adversely influence the track of the Wiimote’s data. We now use rechargeable batteries reset each day prior to running participants.

Skipping: With Python/pygame and DarwiinRemote simultaneously generating a data file (at very high sampling rates, see main paper), there can be occasional skips in the cursor. These skips are not noise – they maintain cursor movement consistent with hand movement. Future work should better integrate modofied DarwiinRemote software with Python presentation software such that only DarwiinRemote captures cursor/accelerometer data, and Python can simply track the status of the experimental trials.
